# Resuscitation after Drowning

**Published:** 1892-09

**Authors:** 


### Resuscitation after Drowning.

At a meeting of the French Academy of Medicine, Laborde (*Munchener Med. Wochensch.*, 1892, No. 28, p. 501) reported the resuscitation from drowning of two cases by separating the jaws and alternately strongly drawing the tongue forward and pushing it backward. The strong traction on the root of the tongue exerts a reflex influence upon the respiratory apparatus, and may be made to conform to the respiratory rhythm.—*Med. News.*

